# The Effect of Using Geosocial Networking Apps on the HIV Incidence Rate Among Men Who Have Sex With Men: Eighteen-Month Prospective Cohort Study in Shenyang, China

**DOI:** 10.2196/11303

**Published:** 2018-12-21

**Authors:** Junjie Xu, Huan Yu, Weiming Tang, Sequoia I Leuba, Jing Zhang, Xiang Mao, Hongyi Wang, Wenqing Geng, Yongjun Jiang, Hong Shang

**Affiliations:** 1 Key Laboratory of AIDS Immunology of National Health and Family Planning Commission Department of Laboratory Medicine The First Affiliated Hospital, China Medical University Shenyang China; 2 Dermatology Hospital Southern Medical University Guangzhou China; 3 Department of Epidemiology University of North Carolina at Chapel Hill Chapel Hill, NC United States

**Keywords:** geosocial networking apps, dating apps, HIV, incidence, homosexuality, male, cohort study

## Abstract

**Background:**

Men who have sex with men (MSM) frequently seek partners through mobile apps (geosocial networking [GSN] apps). However, it is unclear whether GSN apps’ use is associated with the increase in HIV incidence among MSM.

**Objective:**

The aim of this study was to clarify the characteristics of GSN apps’ users and to determine the association and putative mechanisms between GSN apps’ use behavior and HIV incidence.

**Methods:**

We conducted an 18-month prospective cohort study of MSM in Shenyang, China, and the participants were surveyed every 3 months from March 2015 to December 2016. An in-person interview collected information on sociodemographics, GSN apps’ use, recreational drug use, and sexual behaviors. In addition, blood was drawn to test for HIV and syphilis. We used a multivariable Cox regression model to determine possible predictors for increased HIV incidence.

**Results:**

Of the enrolled 686 HIV-negative MSM, 431 (431/686, 62.8%) were GSN apps’ users. Compared with GSN apps’ nonusers, GSN apps’ users were younger; had an earlier age of sexual debut; and in the past 3 months, were more likely to have used recreational drugs, more likely to have had 5 or more casual partners (CPs), more likely to have had group sex with males, and more likely to have had condomless anal intercourse (CAI) with male steady partners (SPs). In addition, 59.4% (256/431) of the GSN apps’ users were willing to accept HIV and AIDS prevention information push services through GSN apps. In total, 19 MSM seroconverted to HIV during the follow-up period; the HIV incidence density rate was 8.5 (95% CI 5.0-13.5) per 100 person-years (PY) among GSN apps’ users and 2.0 (95% CI 0.4-5.8) per 100 PY among nonusers. New HIV infections were independently associated with ever using GSN apps to seek male sexual partners (*P*=.04) and in the past 3 months, using recreational drugs (*P*=.048), having group sex with males (*P*=.01), and having CAI with male CPs (*P*=.02).

**Conclusions:**

GSN apps’ use is associated with higher HIV incidence and may be mediated through recreational drug use and having multiple CPs. Researchers must develop an intervention propagated through GSN apps to reach this high-risk population to mitigate the HIV epidemic in the MSM community.

## Introduction

### Background

Men who have sex with men (MSM) are disproportionately affected by HIV [1-3]. The percentage of MSM with HIV and AIDS in China increased from 13.7% in 2011 to 28.3% in 2015 [4,5]. Understanding the driving forces of this epidemic is essential to provide tailored interventions to MSM.

In the past decade, how MSM seek sexual partners has changed dramatically. In the 1990s, MSM mainly socialized and sought sexual partners in public facilities such as gay bars and bathrooms. Today, with the current popularity of smartphones, tablets, or computers with geosocial networking (GSN) abilities, a large number of GSN apps have been developed and are widely used by MSM. These GSN apps have revolutionized social communication and how MSM seek casual partners (CPs) or multiple sexual partners [6-10]. On average, 36.0% to 63.6% of MSM in the United States [7,11,12] and 40.6% of MSM in mainland China [6] have sought male sexual partners by using GSN apps. Previous research has shown that people who use GSN apps have more sexual partners and more frequent casual (ie, a quick, unplanned encounter without inquiring about the partner’s HIV serostatus) sexual intercourse compared with people who do not, leading to concerns about GSN apps’ use affecting increases in HIV transmission around the world [12-16].

However, the prevalence of HIV and other sexually transmitted infections (STIs) among GSN apps’ users and nonusers is inconsistent with expectations based on the above behavioral differences. A recent meta-analysis concluded that GSN apps’ users compared with nonusers had significantly higher prevalence of STIs, including gonorrhea and chlamydia, but had lower HIV prevalence [16]. Possible reasons for these differences in the prevalence of specific STIs among GSN apps’ users and among nonusers may be related to the designs of these studies. First, in most of these studies, the HIV/STI infection history was developed through the respondents’ self-report [14,17,18], which is subject to recall bias and social desirability bias. Second, most studies were cross-sectional surveys and, thus, only examined the relationship between GSN apps’ use and prevalent HIV infection among MSM. Furthermore, the cross-sectional study design can neither define the time duration of current GSN apps’ use among GSN apps’ users nor can it clarify whether these GSN apps are linked to HIV-related behavioral changes or HIV incidence over time. Finally, compared with using recent HIV infection, using overall HIV infection (includes both recent and established cases) as the outcome of interest does not accurately reflect the effects of GSN apps’ use among MSM. Previous studies have found that among MSM, GSN apps’ users were younger compared with GSN apps’ nonusers, and as this younger population has a shorter duration of potential exposure to HIV, they are expected to have lower HIV prevalence [6,11,14,19,20]. Although 1 recent study reported finding casual sex partners on the internet was an independent risk factor for incidence of HIV infection among MSM in Bangkok, Thailand [21], finding partners through the GSN apps is a more innovative way for MSM to seek sexual partners [6]. No publications have examined the association between using GSN apps and HIV incidence rates among MSM so far. Longitudinal studies of MSM are needed to clarify the HIV incidence among GSN apps’ users over time and to compare the HIV incidence rate among GSN apps’ users with the incidence rate among GSN apps’ nonusers to determine if using GSN apps is contributing to the HIV epidemic among MSM [16].

### Objectives

We conducted an 18-month prospective cohort study among MSM in Shenyang, China, to clarify the characteristics of GSN apps’ users, the association and putative mechanisms between GSN apps’ use behavior and HIV incidence, and their willingness to accept an HIV prevention information dissemination service through a GSN app platform.

## Methods

### Recruitment

Between March 2015 and December 2016, MSM participants in Shenyang, Liaoning province, were recruited through a mixed recruitment method of internet sampling, venue-based sampling, or chain-referral sampling [22]. The inclusion criteria of this cohort were as follows: (1) being 18 years or older; (2) born male; (3) had anal and/or oral intercourse with male partners in the past 6 months; (4) tested as serologically negative for both HIV antibodies and HIV nucleic acid amplification testing (NAAT); and (5) willing and able to provide a written informed consent.

The survey was conducted at The First Affiliated Hospital of China Medical University in Shenyang, China.

### Follow-Up of the Prospective Men Who Have Sex With Men Cohort

All eligible participants were prospectively followed-up at a 3-month frequency. After the initial eligibility interview screening, trained staff interviewed in-person eligible participants in a private counseling room and assigned each participant a unique 6-number identification code to be linked to their laboratory testing results. Venous blood specimens were then drawn and tested for HIV and syphilis. All participants who tested positive for HIV or syphilis received posttest counseling for the infection and referrals to relevant clinics. Each participant received 50 RMB (US $7.4), free condoms, and 1 free lubricant after each completed study visit. Each participant was asked to provide at least two different current methods of contact, and reminder phone calls were made before each follow-up visit.

### Data Collection and Related Measures

Baseline and each follow-up questionnaires repeatedly asked for the following information: (1) sociodemographics, including age, marital status, ethnicity, education, and monthly income; (2) sexual practices in the past 3 months, including number of male sexual partners, how many were steady partners (SPs, sexual activity that takes place between partners in a romantic relationship and usually implies commitment, emotional attachment, or familiarity between sexual partners), how many were casual partners (CPs, affairs like one-night stands or casual sex between males who have little or no knowledge of each other), condom use, and group sex with males (sexual behavior involving more than 2 male participants); (3) recreational drug use in the past 3 months, including using the following types of drugs: poppers (alkyl nitrites), ecstasy, ice (methamphetamine), amphetamine, tramadol, and ketamine; and (4) the names, numbers, and current length of use of GSN apps to seek male sexual partners.

We defined the main outcome of incident HIV infection as seroconversion determined by the presence of HIV antibody during a visit after a previous visit with a laboratory-confirmed HIV-negative serostatus.

We calculated the sample size of MSM participants based on a Cox regression of the log hazard ratio analysis model [23]. When GSN-app-use behavior (X1) is estimated to be .50, the estimated HIV incidence rate (outcome event) is 0.07. We used the parameters of 85% power at a two-sided .05 significance level, an assumed hazard ratio of 3, an SD of X1=0.5, and an *R*^2^ (*R*-squared of X1 with other Xs)= 0.18, and calculated that the smallest sample size was 432 observations. We used PASS (Power Analysis & Sample Size) software version 11 (NCSS, Kaysville, UT, USA) to calculate the sample size.

### Laboratory Testing

After obtaining informed consent at the baseline survey and each follow-up time point, we drew 10 mL of venous blood from each participant to test for HIV and syphilis. HIV-1 antibody screening was performed by enzyme-linked immunosorbent assay, and positive cases were further confirmed through a Western blot test. Specimens that had negative or indeterminate HIV antibody results were further tested using RNA with pooled NAAT (COBAS AmpliPrep, COBAS TaqMan HIV-1 Test, Roche, Germany). Syphilis serology was performed with the rapid plasma reagin (RPR) test (Shanghai Kehua, China), and positive cases were further confirmed by the *Treponema pallidum particle agglutination* assay (TPPA, Serodia, Japan). Participants with plasma positive for both RPR and TPPA were concluded to be currently infected with syphilis.

All related biological tests were conducted in the Key Laboratory of AIDS Immunology of National Health and Family Planning Commission of The First Affiliated Hospital of China Medical University.

### Statistical Analysis

Data were entered twice and checked for accuracy using EpiData Entry software. All data analyses were performed using IBM SPSS (International Business Machines Corporation Statistical Product and Service Solutions) 20.0. MSM who self-reported ever using GSN apps to seek male sexual partners were defined as GSN apps’ users, and the men who self-reported never using GSN apps to seek male sexual partners were considered GSN apps’ nonusers. Comparisons between GSN apps’ users and nonusers and between MSM retained for at least one 3-month follow-up and MSM who withdrew from the cohort were analyzed by chi-square tests. The time of HIV seroconversion was defined as the middle time point between the last laboratory-confirmed HIV seronegative date and the first laboratory-confirmed HIV seropositive date. We measured the follow-up in person-years (PY), and the follow-up spanned from the date of enrollment to either the date of HIV seroconversion or the date of the last follow-up session. We used *a mixed* Cox proportional hazards model to assess cumulative hazard ratios (HRs), both crude (cHR) and adjusted (aHR), for high-risk factors for HIV infection to determine their effects on HIV incidence rates. Time-dependent covariates for the Cox proportional hazards model included over the past 3 months, condom use with male SPs, condom use with male CPs, group sex with males, number of CPs, recreational drug use, and use of GSN apps to seek male sexual partners. The models were adjusted for age, level of education, registered residence, ethnicity, marital status, and monthly income. A two-sided *P* value of less than .05 was considered statistically significant.

### Ethics Statement

This study protocol was reviewed and approved by the Institutional Review Board of the First Affiliated Hospital of China Medical University, with ethical review number of 2011-36. The study protocol, contents, and procedure were explained to each participant before the survey. Written informed consent was obtained from all participants before the interview and blood collection. The procedures in the study were performed in accordance with the study protocol and relevant regulations.

## Results

### Sociodemographic Characteristics of the Participants

**Figure 1:**
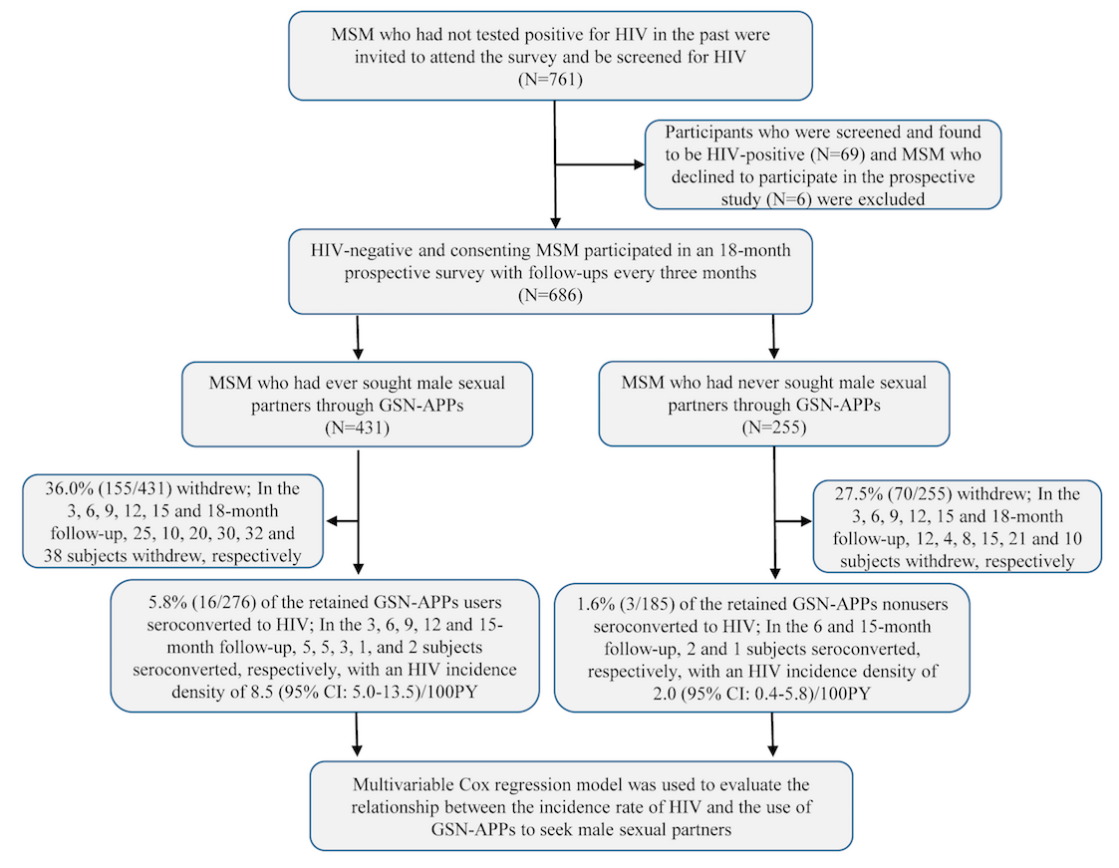
Flowchart of the prospective cohort study examining the relationship between using geosocial networking apps and HIV incidence among men who have sex with men population. MSM: men who have sex with men, GSN: geosocial networking, PY: person-years.

A total of 761 MSM who had no prior positive HIV test were screened for HIV, of which 9.1% (69/761) individuals were detected as HIV-positive and excluded from this study. The HIV-negative MSM were invited to participate in an 18-month prospective cohort study, of which 0.9% (6/692) HIV-negative MSM declined to participate. Thus, a total of 686 eligible HIV-negative MSM were included in this prospective cohort study, of which 431 (431/686, 62.8%) self-identified as GSN apps’ users and 255 (255/686, 37.2%) as nonusers (Figure 1). Table 1 summarizes the sociodemographics, sexual behaviors, recreational drug use, and HIV testing behaviors of the baseline eligible HIV-negative GSN apps’ users and nonusers. Most GSN apps’ users were older than 24 years (294/431, 68.2%), were originally not from Shenyang city (253/431, 58.7%), had college-level education or above (236/431, 54.8%), were ≤20 years at their sexual debut with males (234/431, 54.3%), had male SPs in the past 3 months (250/431, 58.0%), had male CPs in the past 3 months (224/431, 52.0%), and were willing to receive HIV and AIDS prevention information through a push service conducted through a GSN app platform (256/431, 59.4%), but not many GSN apps’ users had been tested for HIV before (113/431, 26.2%). In addition, in the past 3 months, 85.8% (370/431) of GSN apps’ users sought male sexual partners through at least one GSN app, 32.7% (141/431) GSN apps’ users used recreational drugs, 10.9% (47/431) had at least five male CPs, and 8.1% (35/431) had group sex with males. At baseline, 10.4% (45/431) of GSN apps’ users were infected with syphilis.

GSN apps’ users were younger (*P*<.001), had higher proportion of college or above education level (*P*<.001), had higher HIV testing rates (*P*=.02), had higher proportion of recreational drug use (*P*<.001), had higher proportion of group sex with males (*P*=.01), and had higher proportions of having 5 or more CPs in the past 3 months (*P*=.01) compared with its counterpart group.

**Figure 2:**
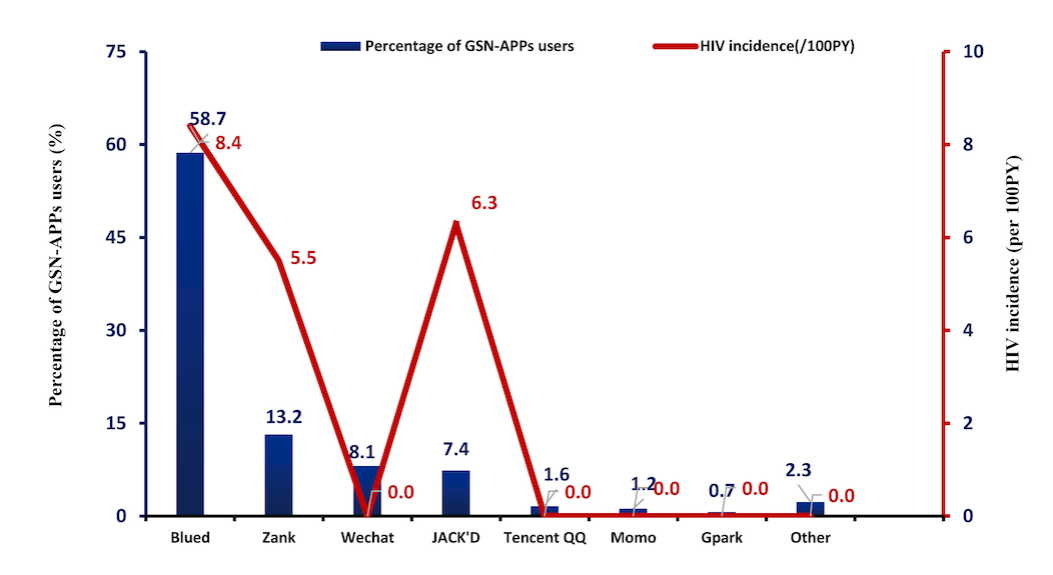
The percentage of men who have sex with men who used geosocial networking apps over the past 3 months to seek male sexual partners and the HIV incidence density for each specific geosocial networking app. GSN: geosocial networking, PY: person-years.

In total, GSN apps’ users reported using mainly 7 types of GSN apps, in which Blued was the most popular GSN app used to seek male partners (403/431, 93.5%), followed by Zank (91/431, 21.1%), WeChat (55/431, 12.8%), Jack’d (51/431, 11.8%), Tencent QQ (10/431, 2.3%), Momo (8/431, 1.9%), and Gpark (4/431, 0.9%). In addition, 3.7% (16/431) of GSN apps’ users used other kinds of GSN apps (Figure 2). In addition, 35.0% (151/431) of GSN apps’ users had once used at least two GSN apps to seek partners, and the median duration for which GSN apps were used was 12 (interquartile range: 4-30) months.

**Table 1:** Sociodemographics and behavioral characteristics of geosocial networking apps’ users and nonusers (N=686).

Characteristics		Total, N (%)	GSN^a^ apps users, n (%)	GSN apps nonusers, n (%)
Total		686 (100)	431 (62.8)^b^	255 (37.2)^b,c^
**Age (years)**				
	18-24	176 (25.7)	137 (32)	39 (15.3)^c^
	>24	510 (74.3)	294 (68.2)	216 (84.7)
**Residency**				
	Shenyang city	269 (39.2)	178 (41.3)	91 (35.7)
	Other	417 (60.8)	253 (58.7)	164 (64.3)
**Ethnicity**				
	Han	570 (83.1)	362 (84.0)	208 (81.6)
	Other	116 (16.9)	69 (16.0)	47 (18.4)
Education level being college or above		319 (46.5)	236 (54.8)	83 (32.5)^c^
**Marital status**				
	Single	466 (67.9)	311 (72.2)	155 (60.8)^d^
	Married or divorced or widowed or cohabiting	220 (32.1)	120 (27.8)	100 (39.2)
**Monthly income**				
	≤3000 RMB/Yuan	392 (57.1)	254 (58.9)	138 (54.1)
	>3000 RMB/Yuan	294 (42.9)	177 (41.1)	117 (45.9)
Currently a university student		79 (11.5)	67 (15.5)	12 (4.7)^c^
Age of sexual debut with males ≤20 years		320 (46.6)	234 (54.3)	86 (33.7)^c^
**Anal sex position**				
	Versatile	287 (41.8)	185 (42.9)	102 (40.0)
	Bottom	264 (38.5)	151 (35.0)	113 (44.3)^e^
	Top	135 (19.7)	95 (22.0)	40 (15.7)
Ever been tested for HIV		160(23.3)	113(26.2)	47 (18.4)^e^
Used recreational drugs in the past 3 months		169 (24.6)	141 (32.7)	28 (11.0)^c^
Had male SPs^f^ in the past 3 months		407 (59.3)	250 (58.0)	157 (61.6)
Had male CPs^g^ in the past 3 months		334 (48.7)	224 (52.0)	110 (43.1)^e^
Two or more male SPs in the past 3 months		68 (9.9)	40 (9.3)	28 (11.0)
Five or more male CPs in the past 3 months		59 (8.6)	47 (10.9)	12 (4.7)^e^
CAI^h^ with male CPs in the past 3 months		114 (16.6)	67 (15.5)	47 (18.4)
CAI with male SPs in the past 3 months		174 (25.4)	94 (21.8)	80 (31.4)^d^
Had group sex with males in the past 3 months		43 (6.3)	35 (8.1)	8 (3.1)^d^
Positive for syphilis at baseline		77 (11.2)	45 (10.4)	32 (12.5)
Willing to accept HIV prevention information push service through GSN apps		259 (37.8)	256 (59.4)	3 (1.2)

^a^GSN: geosocial networking.

^b^These percentages are out of the overall total number of men who have sex with men (N=686). The rest of the percentages are out of the specified group (Total, GSN apps’ users, or GSN apps’ nonusers).

^c^*P*<.001. Statistical significance was set at alpha=.05.

^d^*P*<.01.

^e^The statistical significance of the difference between GSN apps’ users and GSN apps nonusers *P*<.05.

^f^SPs: steady partners (sexual activity that takes places between male partners in a romantic relationship and usually implies commitment, emotional attachment, or familiarity between sexual partners).

^g^CPs: casual partners (a one-night stand or casual sex between males who have little or no history with each other).

^h^CAI: condomless anal intercourse.

### HIV and Syphilis Incidence and Factors Correlated With HIV Seroconversion

During the follow-up period, 36.0% (155/431) of GSN apps’ users and 27.5% (70/255) of nonusers withdrew from the study. In total, 19 MSM who remained in the study seroconverted to HIV during the study period, of which 16 were GSN apps’ users and 3 were GSN apps’ nonusers. The pooled HIV incidence densities were 8.5 (95% CI 5.0-13.5) per 100 PY among GSN apps’ users and 2.0 (95% CI 0.4-5.8) per 100 PY among nonusers and were significantly different from each other (aHR 3.7, 95% CI 1.1-13.1, *P*=.04). Figure 2 showed the percentage of MSM GSN apps users to seek male sexual partners and the corresponding HIV incidence density in each group.

In total, 56 MSM who remained in the study became seropositive to syphilis during the follow-up, of which 34 were GSN apps’ users and 22 were GSN apps’ nonusers. The syphilis incidence densities were similar among GSN apps’ users (17.4, 95% CI 12.0-22.7 per 100 PY) and nonusers (16.1, 95% CI 9.8-22.3 per 100 PY).

**Figure 3:**
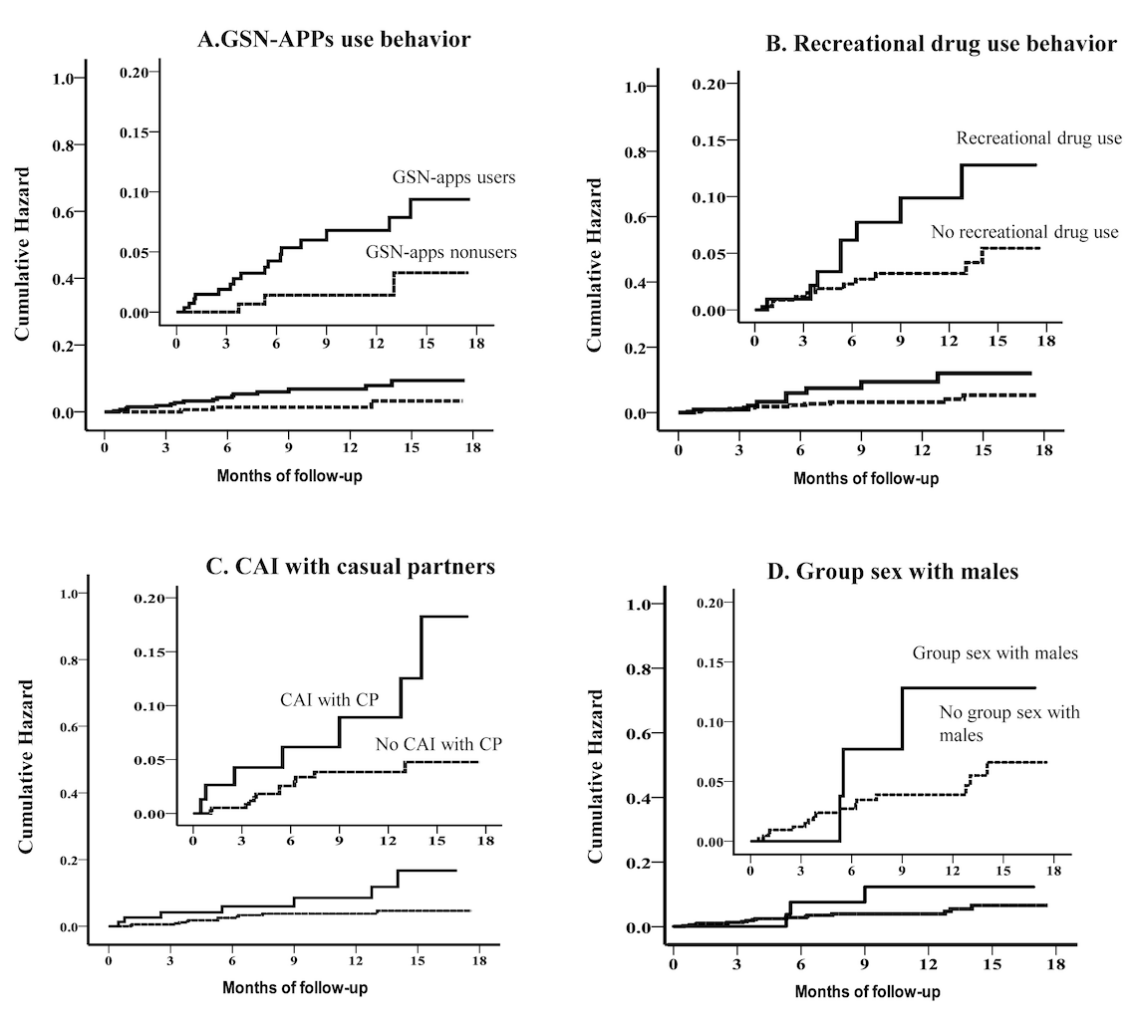
Kaplan-Meier estimates for high-risk factors of HIV seroconversion. GSN: geosocial networking, CAI: condomless anal intercourse, CP: casual partner.

The Kaplan-Meier curves show the differences of the cumulative hazard ratios of HIV seroconversion between GSN apps’ users and nonusers (Figure 3, Panel A), between those who used recreational drugs in the past 3 months and those who did not (Figure 3, Panel B), between MSM who had CAI with CPs in the past 3 months and those who did not (Figure 3, Panel C), and between MSM who had group sex with males in the past 3 months and those who did not (Figure 3, Panel D).

Table 2 contains the HIV incidence among MSM retained in our cohort. Table 3 shows the results of the multivariable Cox regression analysis for predictors correlating with HIV incidence after adjusting for age, level of education, registered residence, ethnicity, marital status, and monthly income. The following characteristics were independently associated with HIV incidence: have ever used GSN apps to seek male sexual partners (i.e. GSN-apps’ users vs nonusers) (aHR 3.7, 95% CI 1.1-13.1, *P*=.04), have used recreational drugs in the past 3 months (aHR 2.6, 95% CI 1.0-6.9, *P*=.048), have had group sex with males in the past 3 months (aHR 4.8,95% CI 1.6-15.0, *P*=.01), and have had CAI with male CPs in the past 3 months (aHR 3.2, 95% CI 1.2-8.4, *P*=.02). The covariate of using GSN apps to seek male sexual partners in the past 3 months had a marginally significant statistical association with HIV incidence (aHR 2.6, 95% CI 0.9-7.9, *P*=.08). In contrast, age of sexual debut (*P*=.90), ever had sexual intercourse with females (*P*=.28), having anal sex position of versatile (*P*=.24) or bottom (*P*=.27) compared with top, having CAI with male SPs in the past 3 months (*P*=.12), the number of CPs (*P*=.30), ever being tested for HIV (*P*=.75), and syphilis infection at baseline (*P*=.67) were not statistically associated with HIV incidence (Table 3).

**Table 2:** HIV incidence among men who have sex with men retained in our cohort in Shenyang (N=461).

Characteristics		Total (N=461^a^)	Incidence, n (%)	Observed person-years (PY)	Incidence rate (per 100 PY)
**Age of sexual debut (years)**					
	≤20	205	9 (4.4)	153.1	5.9
	>20	256	10 (3.9)	181.5	5.5
**Ever had sexual intercourse with females**					
	Yes	246	7 (2.8)	177.2	4.0
	No	215	12 (5.6)	157.4	7.6
**Anal sex position**					
	Versatile	208	7 (3.4)	162.3	4.3
	Bottom	168	6 (3.6)	118.1	5.1
	Top	85	6 (7.1)	54.2	11.1
**Ever been tested for HIV**					
	Yes	95	5 (5.3)	63.9	7.8
	No	366	14 (3.8)	270.7	5.2
**Used recreational drugs in the past 3 months**					
	Yes	141	10 (7.1)	75.3	13.3
	No	320	9 (2.8)	259.3	3.5
**Number of male steady partners in the past 3 months**					
	≥2	51	0 (0.0)	39.4	0.0
	<2	410	19 (4.6)	295.2	6.4
**Number of male casual partners in the past 3 months**					
	≥5	43	3 (7.0)	31.9	9.4
	<5	418	16 (3.8)	302.7	5.3
**Condomless anal intercourse with male casual partners in the past 3 months**					
	Yes	77	7 (9.1)	55.4	12.6
	No	384	12 (3.1)	279.2	4.3
**Condomless anal intercourse with male steady partners in the past 3 months**					
	Yes	120	2 (1.7)	89.2	2.2
	No	341	17 (5.0)	245.5	6.9
**Had group sex with males in the past 3 months**					
	Yes	31	4 (12.9)	28.6	14.0
	No	430	15 (3.5)	306.1	4.9
**Positive for syphilis at baseline**					
	Yes	59	3 (5.1)	47.0	6.4
	No	402	16 (4.0)	287.6	5.6
**Ever used geosocial networking apps to seek male sexual partners (ie, GSN-apps’ users vs nonusers)**					
	Yes	264	16 (6.1)	187.3	8.5
	No	197	3 (1.5)	147.3	2.0
**Used geosocial networking apps to seek male sexual partners in the past 3 months**					
	Yes	239	14 (5.9)	175.5	8.0
	No	222	5 (2.3)	159.2	3.1

^a^The number of GSN apps’ users and nonusers retained to at least one 3-month follow-up visit.

**Table 3:** Multivariable Cox regression analysis of HIV incidence among men who have sex with men retained in our cohort in Shenyang (N=461).

Characteristics of users and nonusers^a^		Crude analysis	Multivariable analysis^b^, cHR^c^ (95% CI)	
			aHR^d^ (95% CI)	*P* value
**Age of sexual debut (years)**				
	≤20	1.3 (0.5-3.3)	1.1 (0.4-2.8)	.90
	>20	Reference	Reference	—^e^
**Ever had sexual intercourse with females**				
	Yes	0.5 (0.2-1.3)	0.6 (0.2-1.6)	.28
	No	Reference	Reference	—
**Anal sex position**				
	Versatile	0.4 (0.1-1.2)	0.5 (0.1-1.6)	.24
	Bottom	0.5 (0.1-1.4)	0.5 (0.1-1.7)	.27
	Top	Reference	Reference	—
**Ever been tested for HIV**				
	Yes	1.5 (0.5-4.1)	1.2 (0.4-3.4)	.75
	No	Reference	Reference	—
**Used recreational drugs in the past 3 months**				
	Yes	2.5 (1.0-6.2)	2.6 (1.0-6.9)	.048
	No	Reference	Reference	—
**Number of male SPs^f^** **in the past 3 months**				
	≥2	N/A^g^	N/A	N/A
	<2	N/A	N/A	—
**Number of male CPs^h^** **in the past 3 months**				
	≥5	1.7 (0.5-6.0)	1.9 (0.6-6.7)	.30
	<5	Reference	Reference	—
**CAI^i^ with male CPs in the past 3 months**				
	Yes	2.9 (1.1-7.4)	3.2 (1.2-8.4)	.02
	No	Reference	Reference	—
**CAI with male SPs in the past 3 months**				
	Yes	0.3 (0.1-1.4)	0.3 (0.1-1.4)	.12
	No	Reference	Reference	—
**Had group sex with males in the past 3 months**				
	Yes	4.3 (1.4-13.1)	4.8 (1.6-15.0)	.01
	No	Reference	Reference	—
**Positive for syphilis at baseline**				
	Yes	1.2 (0.4-4.1)	1.3 (0.4- 4.8)	.67
	No	Reference	Reference	—
**Ever used GSN^j^ apps to seek male sexual partners**				
	Yes	4.1 (1.2-14.2)	3.7 (1.1-13.1)	.04
	No	Reference	Reference	—
**Used GSN apps to seek male sexual partners in the past 3 months**				
	Yes	2.9 (1.0-7.9)	2.6 (0.9-7.9)	.08
	No	Reference	Reference	—

^a^The number of GSN apps’ users and nonusers retained to at least one 3-month follow-up visit.

^b^The multivariable Cox regression model adjusted for age, residence status, ethnicity, education, income, and marital status.

^c^cHR: crude hazard ratio.

^d^aHR: adjusted hazard ratio.

^e^Not applicable.

^f^SPs: steady partners.

^g^N/A: not available.

^h^CPs: casual partners.

^i^CAI: condomless anal intercourse.

^j^GSN: geosocial networking.

### Factors Correlated With Cohort Retention

Compared with MSM who withdrew from the cohort, MSM who were retained to at least one follow-up visit had lower HIV proportion of using GSN apps to seek male sexual partners (57.1% [264/461] vs. 74.2% [167/225], *P*<.01), displayed marginally higher rates of syphilis at baseline (12.8% [59/461] vs 8.0% [18/225], *P*=.06), had marginally higher rates of being older than 20 years at the age of sexual debut (55.5% [256/461] vs 48.4% [109/225], *P*=.08), and had marginally higher proportion of ≥2 SPs in the past 3 months (11.1% [51/461] vs 6.7% [15/225], *P*=.07). There were no statistically significant differences between these 2 groups over the 3 months before the baseline interview in terms of recreational drug use (*P*=.65), having CAI with male CPs (*P*=.93), having CAI with male SPs (*P*=.57), the number of CPs (*P*=.33), having group sex with males (*P*=.48), and having used GSN apps to seek male sexual partners (*P*=.12; Table 4).

**Table 4:** Comparisons of high-risk factors for HIV infection between men who have sex with men who were retained and who withdrew from the follow-up (N=686).

High-risk factors for HIV infection		Retained (n=461), n (%)	Withdrew (n=225), n (%)	Chi-square	*df*	*P* value
**Used recreational drugs in the past 3 months**						
	Yes	141 (30.6)	65 (28.9)	0.2	1	.65
	No	320 (69.4)	160 (71.1)	N/A^a^	N/A	N/A
**Age of sexual debut (years)**						
	≤20	205 (44.5)	116 (51.6)	3.1	1	.08
	>20	256 (55.5)	109 (48.4)	N/A	N/A	N/A
**CAI^b^ with male CPs^c^ in the past 3 months**						
	Yes	77 (16.7)	37 (16.4)	0.0	1	.93
	No	384 (83.3)	188 (83.6)	N/A	N/A	N/A
**CAI with male SPs^d^ in the past 3 months**						
	Yes	120 (26.0)	54 (24.0)	0.3	1	.57
	No	341 (74.0)	171 (76.0)	N/A	N/A	N/A
**Had group sex with males in the past 3 months**						
	Yes	31 (6.7)	12 (5.3)	0.5	1	.48
	No	430 (93.3)	213 (94.7)	N/A	N/A	N/A
**Number of SPs in the past 3 months**						
	≥2	51 (11.1)	15 (6.7)	3.4	1	.07
	<2	410 (88.9)	210 (93.3)	N/A	N/A	N/A
**Number of CPs in the past 3 months**						
	≥5	43 (9.3)	16 (7.1)	0.9	1	.33
	<5	418 (90.7)	209 (92.9)	N/A	N/A	N/A
**Positive for syphilis at baseline**						
	Yes	59 (12.8)	18 (8.0)	3.5	1	.06
	No	402 (87.2)	207 (92.0)	N/A	N/A	N/A
**Ever used GSN^f^ apps to seek male sexual partners**						
	Yes	264 (57.1)	167 (74.2)	18.6	1	<.01
	No	197 (42.7)	70 (25.8)	N/A	N/A	N/A
**Used GSN apps to seek male sexual partners in the past 3 months**						
	Yes	239 (51.8)	131 (58.2)	2.5	1	.12
	No	222 (48.2)	94 (41.8)	N/A	N/A	N/A

^a^N/A: not applicable.

^b^CAI: condomless anal intercourse.

^c^CPs: casual partners.

^d^SPs: steady partners.

^e^GSN: geosocial networking.

## Discussion

### Principal Findings

This 18-month prospective cohort study found that GSN apps’ users had significantly higher HIV incidence compared with GSN apps’ nonusers. We also determined possible mechanisms for how GSN apps’ use causes higher HIV incidence. GSN apps’ users were more likely to participate in group sex with males, have CAI with male CPs, use recreational drugs, and could have used GSN apps to facilitate these risky behaviors that are associated with HIV infection. In addition, 59.4% (256/431) of GSN apps’ users were willing to accept HIV prevention information disseminated through these GSN apps.

### Comparison With Prior Work

Almost all previous peer-reviewed GSN apps–related surveys in MSM have been conducted in the United States and China. In our study, 62.8% (431/686) of MSM participants had used GSN apps at least once to seek male sexual partners, which is similar to the percentage previously published in the United States (36.0%-63.6%) [7,11,12], but slightly higher than a prior study of Chinese MSM (40.6%) [6]. In addition, 85.8% (370/431) GSN apps’ users had sought male sexual partners through GSN apps in the past 3 months; this percentage is higher than the percentage of MSM GSN apps’ users in the United States (56.0%), and the median duration for which MSM use GSN apps to seek male sexual partners in our cohort is similar to that used by MSM in the United States (about 12 months) [17,18]. These results suggest that GSN apps’ use in China is similar to that in the United States; thus, China’s HIV prevention strategies targeting MSM using GSN apps can build on previous experiences of using GSN-APP platforms in the United States to conduct improved novel HIV prevention approaches focused on MSM [24], although the types of GSN apps used by MSM to seek sexual partners may be different.

### Significance of the Study Results

Since the emergence of GSN apps, it is unclear whether their use increases the risk of HIV among their users. It has been speculated that as GSN apps allow for easier access to casual sexual relationships, their use increases the number of sexual partners and, thus, increases the risk of HIV infection [12-16]. In contrast, others have argued that specifically GSN apps’ use does not increase HIV-related high-risk behaviors including CAI [6,19] and, thus, does not increase the risk of HIV infection [11,14,16]. As all previous peer surveys were cross-sectional studies, they were unable to establish temporality and, thus, were unable to draw conclusions on the relationship between putative causes and the outcome of HIV infection. This study showed that among MSM in China, GSN apps’ users have nearly 4 times the HIV incidence rate of nonusers. As this study is a prospective cohort survey, it can not only evaluate whether the HIV incidence rate is linked with GSN apps’ use but can also control the influence of related confounding factors. The multivariable Cox regression model indicated that certain high-risk behaviors are significantly correlated with higher HIV incidence rates after adjusting for potential confounding factors.

In addition, the prospective study allowed for a temporal sequence between putative cause and outcome and, thus, addressed a critical gap in the available literature about GSN apps’ use and new HIV infections among MSM. We were able to determine potential mechanisms underpinning how GSN apps’ use may lead to new HIV infections for its users. We found that GSN apps’ users were more likely to use recreational drugs, have larger numbers of male CPs, and have group sex with males compared with GSN apps’ nonusers. These high-risk behaviors for HIV infection were later confirmed in the multivariable Cox regression analysis to be independent correlates of HIV incidence, and these results were consistent with previous publications [8,10-12,16,25-27]. These results suggest that GSN apps’ use increases the HIV incidence rate among their users through facilitating recreational drug use and higher numbers of sexual partners. Interestingly, we found ever using GSN apps to seek male sexual partners at baseline was an independent significant predictor of HIV seroconversion (*P*=.04), but the covariate of using GSN apps to seek male sexual partners in the past 3 months only had a marginal statistical association with study outcome (.05<*P*<.10). One of the possible reasons for the above difference may be attributed to insufficient efficiency of statistical power for the latter covariate. Statistical power is positively associated with sample size, and the number of participants who ever used GSN apps to seek male sexual partners in this study was just relatively higher than that of participants who used GSN apps to seek male sexual partners in the past 3 months (264 vs 226), which may partly explain the above inconsistence of *P* values. In this study, we used time-dependent Cox regression model to analyze the influence of GSN apps’ use on HIV incidence. The baseline life-time GSN-app using behavior and the GSN app using behavior in past 3 months was set as a fixed covariate and time-dependent covariate, respectively. This data analysis strategy may help public health workers to fully understand the influence of GSN app use behavior within different window periods on HIV seroconversion risk.

Encouragingly, we also found that 59.4% (256/431) of GSN apps’ users in this survey were willing to accept HIV prevention information disseminated through GSN apps. These results have important implications considering the severe social discrimination toward MSM, low sexual orientation disclosure rate, and low HIV testing rate in China [28]. Recently, some social media platforms, including Facebook and Grindr, have collaborated with researchers to disseminate HIV prevention information, promote HIV testing, and link MSM to medical care [24,29]. Thus, future steps include developing interventions circulated through these GSN-APP platforms to reach the target high-risk MSM population to mitigate the HIV epidemic in this community. Further studies need to evaluate the relative impact of HIV prevention interventions disseminated through GSN apps used by MSM compared with traditional facility-based interventions at voluntary counseling and testing clinics or hospitals.

Our study indicated that MSM who use GSN apps compared with nonusers were more likely to be younger than 24 years (31.8% [137/431] vs 15.3% [39/255]) and to be university students (15.5% [67/431] vs 4.7% [12/255]). Currently in China, rates of new HIV infections among young MSM, especially university students, have greatly increased [30]. The Chinese government reported that the number of 15- to 24-year-olds in China who live with HIV more than doubled from 8354 people living with HIV (PLWH) in 2008 to 16,986 PLWH in 2015. Furthermore, the proportion of university students among PLWHs aged between 15 and 24 years increased from 5.8% in 2008 to 19.1% in 2015. Our study results indicate that many young MSM using GSN apps suggest that using these platforms to promote HIV prevention strategies could be effective at targeting young MSM in China.

### Future Studies

The results suggest that GSN apps’ users were significantly associated with higher education levels and higher HIV testing rates compared with nonusers. Studies have shown that people with higher levels of education tend to have higher incomes [31] and, thus, are more likely to have the income needed to purchase expensive smartphones that recognize GSN app software. Less than 50% of Chinese MSM in a prior study got tested for HIV in the previous 12 months [32]; this low HIV testing rate is a serious obstacle in controlling the HIV epidemic [33]. Studies in the United States and in the United Kingdom have shown that promotion of HIV testing can be effectively conducted through GSN app platforms [34,35]. However, currently, there is no published research discussing using GSN app platforms to promote HIV testing among Chinese MSM. The study results support integrating GSN app platforms, in particular Blued, into public health HIV testing promotion strategies to reach MSM.

As there were no significant differences in high-risk sexual behaviors between those who were retained in the study and those who withdrew, it is possible that missing data from those who withdrew from the cohort did not lead to serious bias. Thus, the HIV incidence rate derived from the MSM who were retained to at least one follow-up visit may accurately represent the HIV incidence of the overall recruited MSM population.

### Study Strengths

The study design was a prospective cohort study; this study was conducted among a relatively large sample of MSM and controlled for the influence of many relevant confounders. In addition, it included information on sociodemographics, high-risk behavior for HIV infection, and laboratory testing for HIV and syphilis. Moreover, this study explored possible mechanisms through which GSN apps’ use leads to an increase in the HIV incidence rate; the study results suggested the association between GSN apps’ use and higher HIV incidence rate is possibly mediated through GSN apps facilitating recreational drug use and multiple male CPs.

### Limitations

A potential limitation of this study is reporting bias because of social expectation about the self-reported HIV-related high-risk behaviors, thus leading to underestimation of these behaviors. Second, this study was conducted at a single site, thus limiting extrapolation of its results. Third, participants were not recruited randomly, so the characteristics of participants in this study may not represent well the entire MSM population in Shenyang. Although this study found that ever using GSN apps was correlated with higher HIV incidence rate, GSN apps’ use in the past 3 months was only marginally correlated with HIV incidence. Thus, a larger prospective cohort is needed to further examine the causal relationship between GSN apps’ use and HIV incidence. Finally, approximately 30% of participants withdrew from the prospective cohort during the follow-up period, and the prevalence of syphilis among MSM who withdrew at baseline was marginally lower than that among those who were retained. As syphilis infection can be used as a proxy for unprotected sex, the HIV incidence of Shenyang MSM may be slightly overestimated.

### Conclusions

The GSN apps’ users had higher incidence rates of HIV seroconversion than nonusers, which may be influenced by their higher rates of HIV-related high-risk behavior, including recreational drug use and multiple CPs. Thus, public health workers must collaborate with GSN-app operators to develop an Web-based and offline comprehensive HIV intervention strategy targeting users of these platforms to mitigate the HIV epidemic among MSM.

## Abbreviations

aHR: adjusted hazard ratio
CAI: condomless anal intercourse
cHR: crude hazard ratio
CP: casual partner
GSN: geosocial networking
HR: hazard ratio
MSM: men who have sex with men
NAAT: nucleic acid amplification test
PLWH: people living with HIV
PY: person-years
RPR: rapid plasma regain
SP: steady partner
STI: sexually transmitted infection
TPPA: *Treponema pallidum particle agglutination assay*
